# Progress in Surgery

**Published:** 1898-03-05

**Authors:** 


### Progress in Surgery.

### GENITO-URINARY SURGERY.

(*Continued from page* 381.)

**Urethra.**—Dr. John Brinton (Philadelphia) has submitted his experience of the removal of foreign bodies from the urethra by first placing a finger against the membranous urethra through the rectum, to stop entrance into the bladder, and then exercising manipulation.[1]

Stricture in children may be congenital or traumatic. Dr. Bolton Bangs describes several cases of gonorrhœal origin in young boys, and thinks also that some cases of stricture are due to spreading inflammation to the urethra from balanitis.[2]

The passage of instruments is not without risk. A fatal case is recorded by Zuckerkandl,[3] the patient dying six hours after the operation. Urinary fever is always due to bacterial infection, usually with the bacillus coli. Two fatal cases of septic emphysema are recorded in the *New York Medical Journal*, August 21st, 1897, due to the passage of instruments some days after external perineal urethrotomy. In both cases the Bacillus aërogenes capsulatus was found.

Dr. MacGillivray says that resilient strictures, like an india-rubber ring, are much more easily divided when put on the stretch. He has accordingly devised a urethrotome to this end.[4]

The methodical examination of the urethra and treatment of stricture, as carried out by Professor Guyon, has been reviewed by Mr. Warden.[5] Diagnosis is first made with olivary bougies, and in subsequent treatment with conical olivary bougies; the less force used the more certain are the results.

Dr. Robert Newman (New York) defends the treatment of stricture by electrolysis,[6] using weak currents to act by electrolysis of the cicatricial tissue, not to cauterise.

A new four-bladed urethral dilator has been introduced by Kollman.[7]

Small urethral fistulæ near the meatus are treated by Dr. Chetwood by the local application with a pipette of peroxide of hydrogen.[8]

**Prostate.**—The originator of the modern operation for hypertrophied prostate states that the operation is now much abused. Catheterisation only is necessary if the patient is not very old, retains his sexual power, has sound kidneys, and little residual urine.[9] In worse cases vasectomy may be indicated. The younger and healthier the patient the more one should incline to prostatectomy. In the worse cases, with well-marked cystitis, the risk is greater, but castration should be performed here.

In properly-selected animals, Caspar has shown that double castration always causes atrophy of the prostate. The effect of bilateral division of the vasa deferentia may be either delayed or doubtful. Tying the vas seemed to have no effect on the prostate within four months. Caspar holds, with Pezewalski, that division of the nerves of Cowper accompanying the vas leads to atrophy of the prostate, but not of the testis.[10] The pathology and recent treatment of hypertrophied prostate have been very carefully gone into by F. E. Fremantle, who gives the bibliography and the following conclusions[11]: Castration in childhood arrests development of the prostate, and after puberty causes its diminution, if normal, and its reduction, with relief of symptoms, if hypertrophied. Shrinkage of the prostate is probably due to the removal of some sub-conscious reflex stimulus, normally conveyed to it from the testes by way of the lumbar spinal centre. This is especially suggested by the fact that removal of one testis may cause a corresponding one-sided shrinkage of the prostate. The relief of symptoms is due to relief of congestion and œdema of the prostate.

However, from clinical experience, Lennander says that resection of the vasa gives more encouraging results than castration, and recommends that the surrounding tissue also should be removed so as to divide and destroy the numerous nerve fibres running between the testis and prostate.[12] A patient of Professor Schede (Bonn), without knowing the nature of the operation at all, developed melancholia after vasectomy, from which he completely recovered after five injections of testicular extract. This would tend to confirm the view of an internal testicular secretion.[13]

A case is published where, upon the death of a patient a month after castration, no improvement could be seen in the condition of the prostate.[14] It must be allowed, however, that experiment shows that the results in animals are not absolutely constant. Cabot argues in favour of prostatectomy as compared with castration, and says the mortality is less if anything.

Eugene Fuller has described, purely on clinical grounds, a chronic fibroid contraction of the prostatic fibres encircling the vesical neck.[15] He advises perineal proctotomy for this condition.

Cauterisation of the prostate is reported of favourably. Bottini claims good results from his method of intra-urethral galvano section of the prostate.[16]

Cauterisation through the rectum has been practised by Dr. Negretto with success.[17]

**Gonorrhœa, &c.**—The effects of the gonococcus appear to be due to a toxin produced by the organism which is active after the destruction of the microbe. This explains gonorrhœal inflammations in the absence of living gonococci. A culture with dead cocci is active, but the filtrate appears to be without effect.[18] Epithelium and endothelium are the habitats of the gonococcus, the constitutional effects being toxic. With regard to bilateral epididymitis it appears that sterility follows in about one-third of the cases.

Gonorrhœa in the female is now known to affect chiefly the urethra, and next in order the cervix uteri. The naked eye appearance is no criterion to the disease, as the diplococci are often present in a perfectly clear fluid.[20] D'Aulnay holds that the local effects are due to the action of the gonococcus on the mucous membrane, but that the constitutional effects are usually due to the toxin produced. The blood, kidneys, nervous system, skin, and joints are chiefly affected; but the micro-organisms themselves sometimes pass into the tissues, leading to local and metastatic suppurations when combined with other organisms.[21] The gonococcus is now recognised as one of the causes of endocarditis. A husband with gonorrhœa infected the wife, who soon died of vegetative endocarditis, and in the vegetations the gonococcus was found.[22]

Several articles have lately appeared upon the treatment of gonorrhœa.[23] Airol (oxy-iodo-gallate of bismuth), argonin (casein combined with silver nitrate), ichthyol, citrate of silver, and others have been credited with success. Large douches of a weak solution of potassium permanganate are recommended. Dr. George Howland thinks that in the permanganates of potassium and zinc, formalin, and nitrate of silver we have all that can be desired. The method of free irrigation is gaining favour, and, as shown by Dr. Valentine, this may be performed without passing a catheter.[24]

**Syphilis.**—Hard chancres are sometimes multiple. Sack describes a case in which there were as many as 15. It must be noted that the patient was one who scratched himself a great deal, and that 11 of the sores appeared in the sites of healed abrasions.[25] Dr. Lesné exhibited a case with four typical chancres.[26]

Evidence is now certain that hereditary syphilis *may* occur to the third generation. Dühring records five such cases. He also finds that healthy children born of syphilitic parents possess a short immunity from acquired syphilis or show insidious syphilitic manifestations.[27] The question of transmissibility to the third generation has been very fully gone into by Dr. George Ogilvie in the *British Journal of Dermatology* for 1897. The question must still be regarded as uncertain.

Of interesting cases lately published the following may be mentioned: Syphilitic osteitis of humerus and femur in a child of 11 months,[28] symmetrical exostoses of hereditary syphilitic origin,[29] fractures in syphilis,[3] tertiary syphilitic epididymitis.[31]

In the discussion on the treatment of syphilis at the 1897 annual meeting of the British Medical Association, Professor Whitla laid great stress on taking the weight of the patient to estimate the effect of the two great drugs, mercury for the disease, and iodides for the complications. Dr. Leslie Phillips shows the desirability of occasionally varying the mercurial formula, watching for mercurialism, and particularly treating locally all the local manifestations.[32]

As regards the serum treatment of syphilis Professor Boeck speaks in favour of it to some extent.[33] It appears that the serum from a case of tertiary syphilis is the more active, and this is confirmed by the researches of Dr. Vïeviorovski.[34]

[1] N.Y. Med. Record, Ap. 10, 1897. [2] N.Y. Med. Record, Ap. 10, 1897. [3] Epit. B.M.J., May 15, 1897. [4] Lancet, July 17, 1897. [5] Glas. Med. Jour., Sept., 1897. [6] N.Y. Med. Record, March 27, 1897. [7] N.Y. Med. Record, Aug. 28, 1897. [8] N.Y. Med. Record, July 3, 1897. [9] Lancet, May 8, 1897. [10] Berliner Klinische Wochenschrift, 1897, No. 27. [11] Guy's Hosp. Gazette, March 13, 1897. [12] Epit. B.M.J., July 17, 1897. [13] Med. Week, May 7, 1897. [14] Internat. Med. Magazine, Aug., 1897. [15] Med. Record, July 3, 1897. [16] Archiv. für Klin. Chir., 1897, Band liv., Heft 1. [17] Gazz. degli Osped., Dec. 27, 1896. [18] Medical Week, July 30, 1897. [19] Internat. Med. Mag., Oct., 1898. [20] Finger, Wien Klin. Woch, 1897, No. 3. [21] Epit. B.M.J., July 17, 1897. [22] Deutsche Medicinische Woch, May 13, 1897. [23] Clinical Jour., July 21, 1897; Medical Bulletin, April, 1897. [24] N.Y. Med. Record., June 5, 1897. [25] Amer. Jour. Med. Sci., Nov., 1897. [26] Med. Week, July 16, 1897. [27] Epit. B.M J , Sept. 4, 1897. [28] N.Y Med. Jour., May 29, 1897. [29] Epit. B.M.J., May 15, 1897. [30] Med. Week, March 5, 1897. [31] Annals of Surgery, March, 1897. [32] Brit. Journ. Dermatol., May, 1897. [30] Med. Chronicle, March, 1897. [34] See N.Y. Med. Rec., June 5, 1897.

